# Current antimicrobial use in farm animals in the Republic of Ireland

**DOI:** 10.1186/s13620-020-00165-z

**Published:** 2020-06-26

**Authors:** Hannah Martin, Edgar Garcia Manzanilla, Simon J. More, Lorcan O’Neill, Lisa Bradford, Catherine I. Carty, Áine B. Collins, Conor G. McAloon

**Affiliations:** 1 School of Veterinary Medicine, University College Dublin, Belfield, Dublin 4, Ireland; 2 Pig Development Department, Teagasc Animal and Grassland Research and Innovation Centre, Moorepark, Fermoy, Co. Cork, Ireland; 3 Centre for Veterinary Epidemiology and Risk Analysis, School of Veterinary Medicine, University College Dublin, Dublin, Ireland; 4 Department of Agriculture, Food & the Marine, Agriculture House, Kildare St, Dublin 2, Ireland

**Keywords:** Antimicrobials, Usage, Food animals, Ireland

## Abstract

Antimicrobial resistance has been recognised as one of the most difficult challenges facing human and animal health in recent decades. The surveillance of antimicrobial use in animal health plays a major role in dealing with the growing issue of resistance. This paper reviews current data available on antimicrobial use in farmed animals in the Republic of Ireland, including each of the major livestock production sectors; pigs, poultry, dairy, beef and sheep. A systematic literature search was conducted to identify relevant published literature, and ongoing research was identified through the network of authors and searches of each of the research databases of the main agriculture funding bodies in Ireland. The varying quantities and quality of data available across each livestock sector underlines the need for harmonisation of data collection methods. This review highlights the progress that has been made regarding data collection in the intensive production sectors such as pigs and poultry, however, it is clear there are significant knowledge gaps in less intensive industries such as dairy, beef and sheep. To comply with European regulations an antimicrobial data collection system is due to be developed for all food-producing animals in the future, however in the short-term surveillance studies have allowed us to build a picture of current use within the Republic of Ireland. Further studies will allow us to fill current knowledge gaps and build a more comprehensive overview of antimicrobial use in farm animals in Ireland.

## Introduction

Antimicrobials (AM) are a vital tool in both human and animal health. However, overuse of AM can contribute to the development of antimicrobial resistance (AMR) [1] which has been recognised as a global issue for human and animal health in recent decades. Antimicrobials are used in significant quantities in agriculture and this use can lead to the development of AMR in bacteria in animals [2], which may be transmitted to humans through direct contact with animals, the food chain or the environment [3]. Many of the same AM that are used in humans are also used to treat infections in animals, including AM listed by the World Health Organisation (WHO) as ‘critically important’ (CIAs) for humans [4]. The overuse of CIAs may result in the loss of their efficacy due to development of AMR, therefore once-treatable human infections may become potentially fatal.

Since 2005, the WHO has produced a regularly updated list of all AM currently used for human medicine (most are also used in veterinary medicine), grouped into 3 categories based on their importance to human medicine [4]. Within the European Union (EU), the European Medicines Agency (EMA) have published a list categorising AM for use in animals prepared by the Antimicrobial Advice Ad Hoc Expert Group (AMEG). The European classification now comprises four categories, from A to D: Avoid, Restrict, Caution and Prudence, respectively. The categorisation of AM classes for veterinary use in the EU, with examples of active substances per class, is available on the EMA’s website [5]. The categorisation is intended as a tool to support decision-making by veterinarians on which AM is appropriate to use to reduce the threat of AMR development.

In 2015, the WHO published the Global Action Plan on AMR [6] including five strategic objectives to combat the threat of AMR to both human and animal health. A key objective of the WHO Global Action Plan on AMR focuses on strengthening knowledge of AMR and antimicrobial use (AMU) through surveillance [6]. Reliable data on the quantities of AM used are needed to benchmark usage, observe trends over time and to monitor the response to any interventions to reduce use [7]. Within the EU, the EMA promotes the prudent use of AM in humans and animals, and the collection of AMU data. EU member states are encouraged to record and report usage data in both human and animal health so that the impact of policy change may be monitored. Additionally, new EU Veterinary Medicines Regulations (EU) 2019/6 [8] will make it a requirement for AM prescription data on all food-producing animals to be collected in a national AM consumption database from January 2027.

Several EU member states, such as Belgium [9], Denmark [10], France [11], Sweden [12], the Netherlands [13] and the United Kingdom (UK) [14] publish annual reports on the consumption of veterinary AM at a national level. The introduction of surveillance programmes, based on routine, farm-level collection of usage data, has allowed many of these countries to make significant progress in reducing their AMU. In the Republic of Ireland, national usage data is not yet collected at farm level. However, the Department of Agriculture, Food & the Marine (DAFM) introduced an AM database collection system for commercial pig herds in November 2019, with plans to develop similar systems for other livestock sectors to comply with new EU legislation [8].

In 2017, Ireland published the National Action Plan on Antimicrobial Resistance 2017–20 (*i*NAP) [15]. This report outlines the steps needed to reduce the threat of AMR from the agricultural sector, including the collection of AMU data. As yet, there is no real-time collection of AMU data in Ireland. However, there are several examples of research conducted in Ireland to quantify AMU across the various livestock sectors. An all-island review of current AMU was not feasible as data from Northern Ireland are generally aggregated with data from Great Britain. Therefore, the purpose of this study was to review current AMU in the Republic of Ireland across and within the range of agricultural sectors, based on data that are available, to review current methodology that is being used to quantify AMU, and to identify gaps in sector-specific AMU that are not currently addressed in published or ongoing research.

## Materials and methods

### Systematic literature review of AMU

A systematic literature search was conducted using the databases PubMed and CAB Direct to identify relevant articles. The literature search was limited to articles published during 2010–2019. An initial search was first carried out for literature in all animal production sectors. Next, separate, independent searches were performed for each of the major production sectors; pigs, poultry, dairy, beef and sheep, with the final search results pooled. The search terms used were:

1. (((Antimicrobial) OR Antibiotic) AND Ireland) AND Animal Production
2. (((Antimicrobial) OR Antibiotic) AND Ireland) AND Pig OR Swine
3. (((Antimicrobial) OR Antibiotic) AND Ireland) AND Poultry OR Broiler OR Chicken OR Layer
4. (((Antimicrobial) OR Antibiotic) AND Ireland) AND Dairy OR Cattle OR Calf
5. (((Antimicrobial) OR Antibiotic) AND Ireland) AND Beef
6. (((Antimicrobial) OR Antibiotic) AND Ireland) AND Sheep

The titles of search results were screened by the first author. The criteria for selecting an article based on the title was the inclusion of the following phrases or ideas related to them: antibiotic use or therapy, antimicrobial use or therapy, or in-feed medication. Articles with relevant titles were exported and their abstracts were screened. Potentially eligible full-text manuscripts were then reviewed, and irrelevant articles were excluded. Inclusion criteria required that the articles dealt with one of the following categories of information: quantifying antimicrobial use, describing antimicrobial usage patterns, or collection of antimicrobial usage data.

### Ongoing AMU research

A search was conducted to identify ongoing research projects in the area of AMU in animal production within Ireland. Searches were conducted of each of the research databases of the main agriculture funding bodies in Ireland.

#### DAFM

The Department of Agriculture, Food & the Marine (DAFM) funds projects under three competitive funding research programmes for agriculture, food and forestry. The summary details and final reports (if available) of current and ongoing projects are published on their website along with the names of the principal investigators [16]. A search was carried out of the DAFM website to identify relevant projects.

#### Safefood

*safe*food is the Irish Food Safety Promotion Board. *safe*food funds research on the island of Ireland in the areas of food safety, food hygiene, nutrition and healthy eating, and publishes information on funded projects on their website [17]. A description of the project is given, and any associated published reports, digital resources and peer-reviewed publication are listed. A search was conducted of the research portfolio on the *safe*food website, and project titles were then screened for relevance.

#### Teagasc

Teagasc, Ireland’s national Agriculture and Food Development Authority provides training and advisory services to the agriculture and food industry as well as undertaking research. Teagasc provides funding for research in four main areas: ‘Animal and Grassland Research and Innovation’; ‘Crops, Environment and Land Use’; ‘Food’ and, ‘Rural Economy and Development’ [18]. A search was carried out of the Teagasc website to identify relevant projects funded through internal Teagasc funds and the Teagasc Walsh Fellowship fund. In addition, a search was conducted of the Teagasc TResearch publications. Teagasc TResearch is a technical science publication that communicates the scientific work being undertaken in Teagasc and collaborating research institutions. The search was limited to articles published in the last 10 years, 2010–2019. The same inclusion criteria as used in the systematic literature search were applied here.

### Antimicrobial sales

Since 2009, the Health Products Regulatory Authority (HPRA; known as the Irish Medicines Board (IMB) prior to 2014) has produced an annual report on sales of veterinary AM in the Republic of Ireland. The report collates data on AM sales for the previous year provided to the HPRA by the marketing authorisation holders that market veterinary AM. The data is submitted to the EMA as part of the European Surveillance of Veterinary Antimicrobial Consumption (ESVAC) project. A search was carried out of the HPRA website to identify reports from recent years.

## Results

### Background

#### Systematic literature review of AMU

**Fig. 1:**
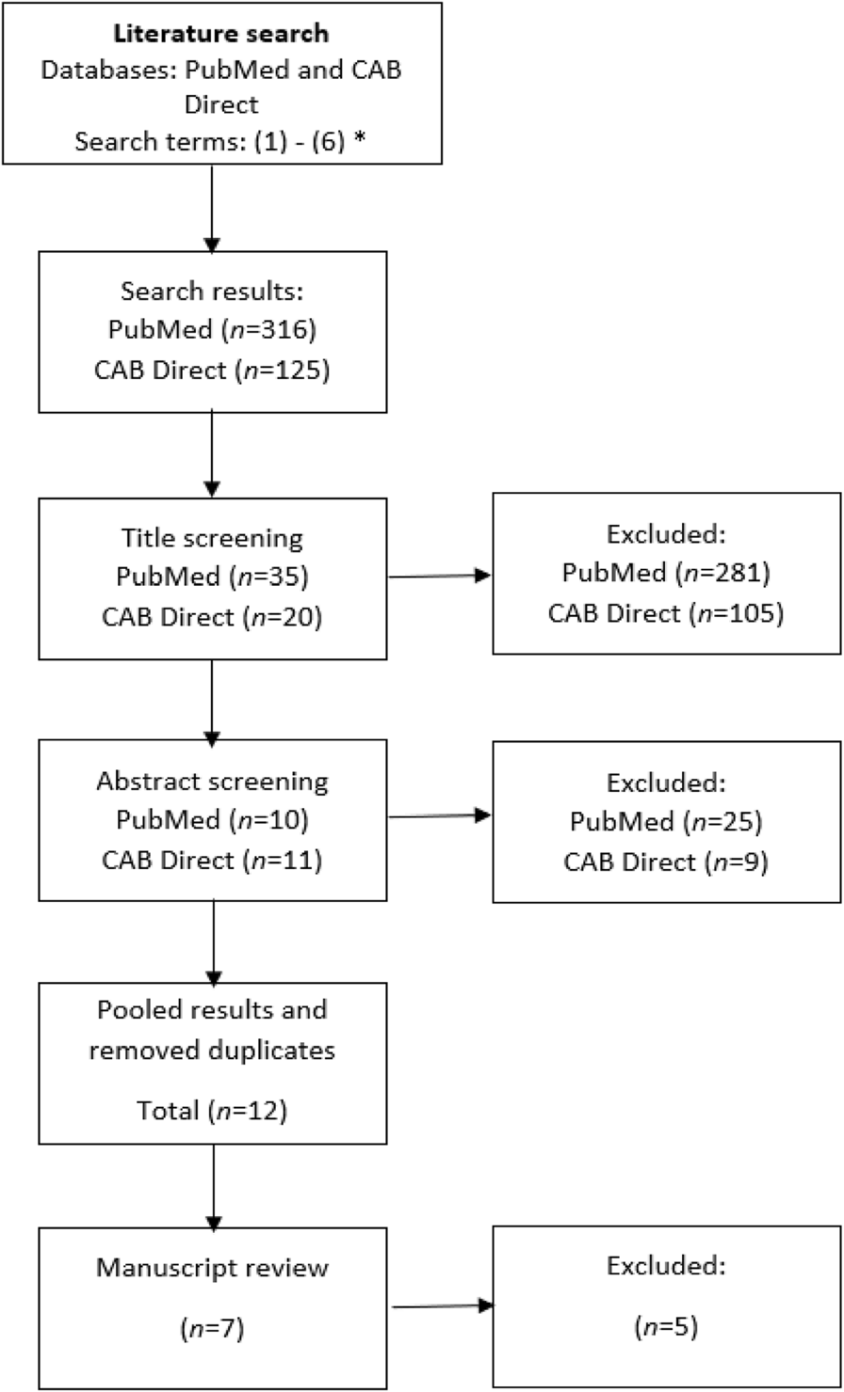
Flow diagram of the database search process for identifying relevant peer-reviewed publications

Figure 1 illustrates the search process for peer-reviewed published literature. Three articles discussing AMU in Irish pigs were identified from the database search. These articles discuss patterns of AMU and the effects on AMR, health and welfare [19–21]. Three articles were available on AMU in the dairy sector [22–24]. One article was available for the beef sector discussing AMU in calves [25]. No peer-reviewed data were available for current AMU in the poultry or sheep production sectors.

#### Ongoing AMU research

The search for ongoing AMU research identified work that has been carried out in the pig and poultry sectors under the Antimicrobial Use and Resistance in Animal Production (AMURAP) project funded by DAFM [26]. This project aims to gain a deeper understanding of the current AMU on commercial Irish pig farms and broiler crops. AMURAP is on-going and has to date focused on; the classes and quantities of AM used, and the patterns of use. Farm-level data has been recorded for the broiler sector as part of the AMURAP project, although this data has not yet been published [27]. The search also revealed projects funded by Teagasc and DAFM such as WELPIG and PATHSURVPIG which have explored the relationship between AMU, respiratory disease and welfare in pigs [19, 20]. Within the dairy industry, data on intramammary AMU for the years 2016–2019 have been analysed and are currently under review.

#### Antimicrobial sales

Ten reports on the consumption of veterinary AM were available on the HPRA website. Reports were available for the years 2009–2018. The reports provide a figure for the total tonnage of veterinary AM sold that year and breaks this figure down into AM classes and pharmaceutical form. Reports from the last 5 years were analysed [28–32].

### National AMU

**Fig. 2:**
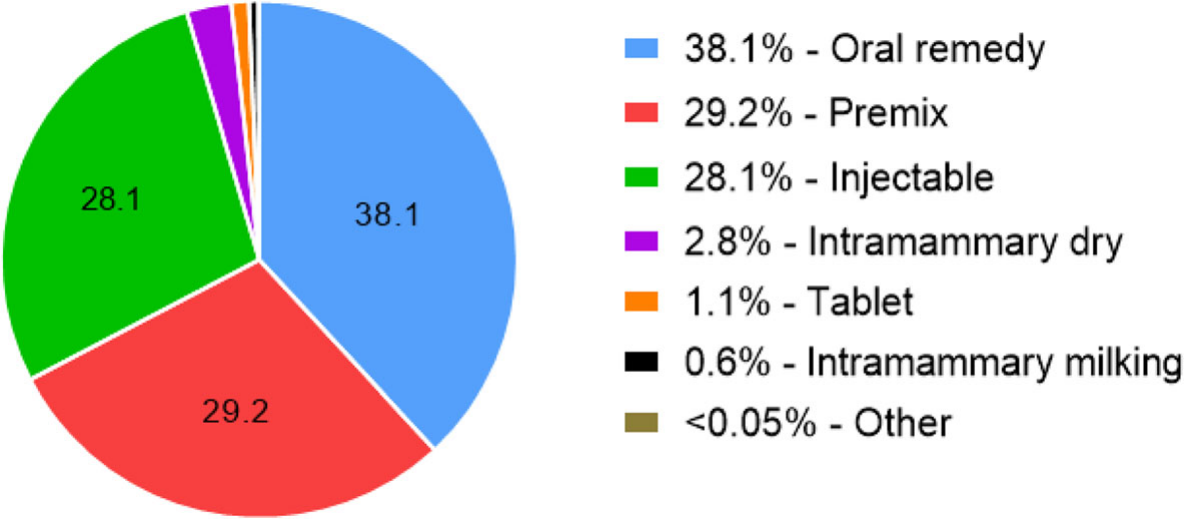
Pharmaceutical form breakdown of veterinary antimicrobials sold in Ireland in 2018 [28]

The total amount of veterinary AM sold in Ireland in 2018 was 99.4 t [28]. Figure 2 depicts the breakdown of AM supplied to the market in pharmaceutical form sold for 2018.

**Fig. 3:**
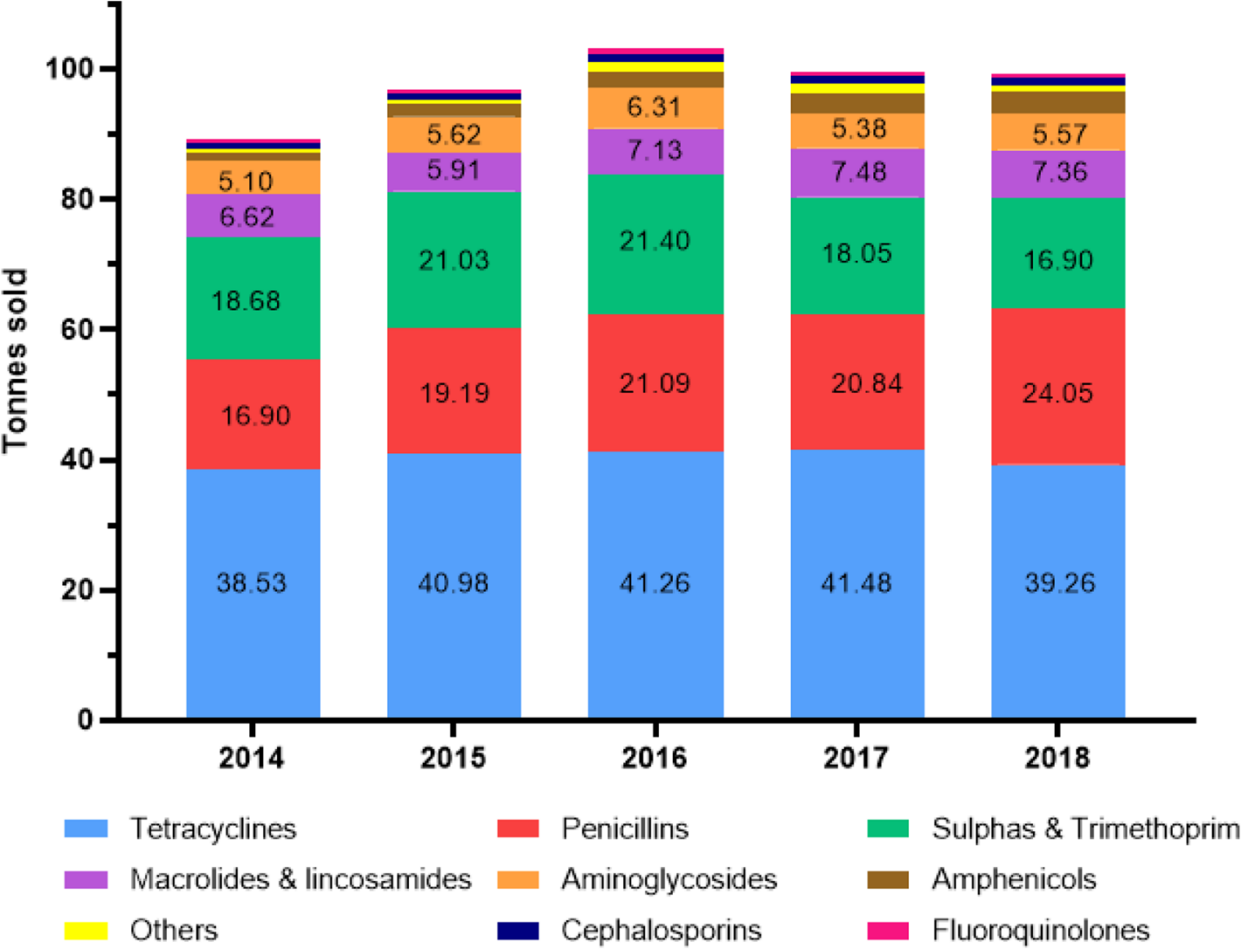
Distribution of sales (based on tonnes sold) of veterinary antimicrobials supplied from 2014 to 2018 in Ireland, by AM class [28–32]

Over the last 5 years the sales of veterinary antimicrobials have remained between approximately 90–100 t (Table 1). The distribution of sales in classes of AM sold for the years 2014–2018 are illustrated in Fig. 3. The classes of AM sold have not changed substantially over the last 5 years. The sales of fluoroquinolones, classed as Category B (‘Restrict’) by the EMA, have remained relatively constant over this period, while the sales of the Category C (‘Caution’) macrolides and Category B 3rd & 4th generation cephalosporins have increased (Table 1).

**Table 1:** Sales (tonnes sold) of veterinary antimicrobials including 3rd & 4th generation cephalosporins, fluoroquinolones & macrolides for the years 2014–2018 [28–32]

Year	2014	2015	2016	2017	2018
**All AM classes**	89.4	96.9	103.4	99.7	99.4
**3rd & 4th gen. Cephalosporins**	0.24	0.22	0.25	0.30	0.33
**Fluoroquinolones**	0.69	0.79	0.94	0.85	0.84
**Macrolides**	6.26	5.58	6.58	7.17	7.07

The sales data collected by the HPRA is submitted to the EMA under the ESVAC project, and the EMA publishes an annual report on the sales of veterinary AM in Europe, the most recent report published in 2019 is based on data from 2017 submitted by 31 European countries [33]. The amounts of veterinary AM sold are reported in tonnes of active ingredient and in mg of active substance per Population Correction Unit (PCU) where PCU is an estimate of weight at treatment defined for each species. PCU is a theoretical unit of measurement developed by the EMA in 2009 [34]. The estimated PCU for Ireland (in 1000 t) of the population of food-producing animals for 2017, was 2114 [33]. That year, Ireland’s annual sales of veterinary AM was 99.7 t of active ingredient or 46.6 mg/PCU [33]. In terms of Category B AM, fluoroquinolones and 3rd & 4th generation cephalosporins had figures of 0.4 mg/PCU and 0.1 mg/PCU, respectively, in Ireland for 2017.

### AMU by farm sector

#### Pigs

The AMURAP project analysed a range of data to quantify AMU in the pig sector over a 12-month period in 2016. The total consumption of AM by weight of active ingredient was 14.5 t for the sample population; this sample represented one-third of the national population of pig herds, suggesting the AMU for the population of approximately 140,000 to 150,000 sows would be approximately 43.5 t of active ingredient. The total AMU for the sample farms combined was 161.9 mg/PCU [35].

The largest group of AM used (by weight of active ingredient) were tetracyclines (55.8%), followed by sulphonamides and trimethoprim (25.2%), macrolides (9.3%) and penicillins (7.8%), the remainder was made up of other AM classes [36]. The use of AM categorised as Category B was low in the study farms, however, the number of farms exposed was high, especially for fluoroquinolones.

In terms of routes of administration, 97.5% of AM were administered orally; 89.2% in medicated feed, 7.2% in water, 1.1% in oral powder and 0.03% in oral doses. The remaining 2.5% was administered through injectables [36]. The large proportion of AMU attributed to oral remedies indicates AM are most frequently applied as group treatments. Analysis of AMU in medicated feed showed that 75.5% of all AM used in pig production are administered to pigs in the post weaning stages of production (4–13 weeks old).

Patterns of AMU in medicated feed on pig farms in Ireland are also described in a separate study carried out by Gibbons and colleagues, although this study does not provide quantities of AM used [21]. The findings are in agreement with those of O’Neill and colleagues [36] in that AM were frequently administered in the post weaning stages.

#### Poultry

Due to the confidentiality agreement between the researchers and the producers who provided the data it was not possible to report the quantities of AM used by the broiler crops in the AMURAP project study [27] in this review. The classes of AM used included penicillin (amoxicillin) and potentiated sulphonamides (sulfamethoxazole, sulfadiazine, and trimethoprim). There was no recorded use of AM classed as Category A, B or C in those crops.

The research analysed the number of diagnoses that received a prescription for an AM and categorised the results by diagnosis group. Nearly all diagnoses of young chick diseases received a prescription and nearly half of the diagnoses for intestinal disease received a prescription; almost half of all AMU occurred in the first week of life [27].

#### Dairy cattle

No data were available to estimate the overall quantities of AM used in the dairy industry. AMU in dairy calves representing calves reared in dairy-calf-to-beef systems has been studied and is discussed under beef cattle [25]. At a national level, only intramammary AMU has been described. More and colleagues used national sales and prescription data to describe trends in both intramammary Dry Cow Therapy (DCT) and intramammary Lactation Therapy (LT) AMU in dairy herds in Ireland [22, 23].

Sales of DCT intramammary products increased from 2003 to 2015 by approximately 106,000 tubes per year from a baseline of 2,250,000 [23]. The number of teat sealants sold also increased, indicating that farmers were using teal sealants in conjunction with, rather than as an alternative to using intramammary AM products. However, work by McAloon and colleagues (in review) has demonstrated a decline in DCT AMU between 2015 and 2019. In addition, the dairy industry has made a concerted move towards Selective Dry Cow Therapy (SDCT) under the umbrella of the CellCheck mastitis control program of Animal Health Ireland [37]. In contrast, there was a decrease in sales of LT intramammary products of approximately 26,000 tubes per year from 2003 to 2015 [23].

The classes used in intramammary AM products for DCT included aminoglycosides, cephalosporins and penicillins [23]. The use of 1st generation cephalosporins, categorised by the EMA as Category C in DCT products has increased steadily to a figure of 481 kg (quantity of active substance) in 2015 from a baseline of 147 kg in 2003 [23]. In LT tubes, the classes of AM used included aminocoumarin, aminoglycosides, cephalosporins, lincosamides, penicillins and sulphonamides. Macrolides and tetracyclines have not been present in LT intramammary products since 2005 and 2009, respectively. However, there has been continuous use of Category C 1st generation cephalosporins, and Category B 3rd and 4th generation cephalosporins in LT intramammary products over the period studied. Whilst there was an overall decrease in the quantity of active substance in LT intramammary tubes sold over the years, there was an increase in the sale of products containing 4th generation cephalosporins, with over 3 times as many 4th generation cephalosporins sold in 2015 than in 2003 [23].

#### Beef cattle

No data were available to estimate the overall quantities of AM used in the beef industry. The only research published to date in this area has quantified use in 0–6-month-old beef and dairy calves and the contribution of adult beef cattle has not yet been studied [25, 38]. Earley and colleagues studied AMU in the two main calf-rearing systems in Ireland, suckler calf-to-beef and dairy calf-to-beef. The average AMU for suckler beef calves from birth to six months was 7.25 mg/PCU with approximately two-thirds of that given in the first month of life and with 20.4% of suckler beef calves treated with AM for at least one disease event [25]. Dairy calves had an average AMU of 7.11 mg/PCU and 14.8% of dairy-bred calves were treated with AM at least once.

The AM prescribed most for dairy and suckler-beef calves during the study period were penicillin, mainly amoxicillin, tetracyclines mainly oxytetracycline, amphenicols (florfenicol) and fluoroquinolones (enrofloxacin and marbofloxacin) [25]. Fluoroquinolones, which are classed as Category B antimicrobials by the EMA, ranked highest in terms of Treatment Incidence (TI) across all calves in this study.

#### Sheep

No data were available to estimate the quantities of AM used in the sheep industry, either at an overall level or within a particular age group or specific use.

## Discussion

This study highlights a significant deficit in the knowledge of AMU at sector-level in farmed animals in the Republic of Ireland. Currently, the only national-level AMU data is derived from sales data of veterinary AM medicines, and there are issues with sales data that need to be considered. Firstly, the figures are not corrected for population size, therefore fluctuations in sales may reflect changes in the national herd size. Furthermore, sales do not necessarily equate to on-farm use of AM and these figures do not consider AM which are held in stock. Additionally, the sales data includes sales of veterinary AM for use in companion animals, which may account for a larger proportion than expected. However, accepting these issues, some useful interpretations may be taken from the data. For example, while most pharmaceutical forms are used across sectors and therefore cannot be attributed to a specific industry, it is possible to relate the sales of intramammary products to the dairy sector. Furthermore, combining sales data with findings from research studies can help identify major gaps and possible attribution of sales data. For example, it was previously suspected that the cattle industries contribute little to the overall quantity of AM used when compared to more intensive industries such as pigs and poultry. However, further to recent research in the pig and poultry industries, up to 19 t of oral veterinary AM sold nationally are not accounted for in these industries [27, 36, 39]. It is possible that a large proportion of the approximate 19 t of oral AM sold may be attributed to use in the cattle industries for the treatment of sick calves. While the use of oral AM in calves in Ireland has been mentioned in literature [40], as yet no studies have investigated the quantities used for this purpose. Furthermore, the contribution of oral AMU in laying hens has not been considered. Sector-level data collection is needed to accurately attribute sales of oral AM to use in a specific industry.

The collection of sector-level data will add value to the sales data gathered, currently in Ireland, veterinary AM consumption is categorised by antimicrobial classes and pharmaceutical form sold [28]. In contrast, in countries such as Denmark and the Netherlands, sector-level data collection has allowed the distribution of AM consumption to be categorised by the main species [10, 13]. Categorising use by species has allowed these countries to identify the main industries driving AMU in the country and therefore implement targeted reduction strategies.

While AMU data is not routinely collected for all species at sector-level in Ireland surveillance studies have provided insights into the quantities of AM used at sector-level, however, the quality of data available varies. Across the sectors, the pig industry has collected the most robust data on overall use, including quantities used, classes used and routes of administration. The quality of the data collected has allowed for estimates of overall use to be calculated and a large proportion of national sales to be accounted for. In 2016, the pig sector was estimated to have used 43.5 t of the veterinary AM sold [36], being 42% of total veterinary AM sold that year (103.4 t) [31].

Unlike the pig industry, less intensive industries have not gathered sufficient data to calculate a figure of overall use within the sector. Within the dairy industry, the data collected on intramammary AMU by More and colleagues has provided invaluable information on the use of intramammary products [22, 23]. However, a major limitation of this research is that only intramammary AM are discussed, the contribution of injectables or intrauterine AM are not considered. In a study conducted in the UK by Hyde and colleagues evaluating AMU on British dairy farms, intramammary products only made up 12.5% of AM used or sold, whereas injectables made up 78.1% [41]. The lack of data available, both at sales or prescriber level or indeed at farm level on non-intramammary AMU in the dairy sector is a challenge that will have to be overcome in order to better quantify AMU within this industry.

In addition to the forms of AM measured, the population studied can influence the quality of the AMU data collected. In research in the pig and poultry industries, the sample population included all age groups on the study farms [27, 36], thus allowing for a figure of overall use within the sector to be calculated. In other sectors, the contribution of each age group to overall use has not been considered. Studies which evaluate usage across all age groups on the farm glean a more holistic view of AMU across the sector, the exclusion of certain groups may affect the comparability of the data.

The comparability of animal production AMU data is a current issue in Ireland. Although some AMU data has been collected in most livestock species, it is difficult to make cross species comparisons due to the difference in metrics used to quantify use. The research studies included in this review used varying methods for quantifying AMU such as mg/PCU, DDDvet and DCDvet, or Treatment Incidence (TI). There are benefits to each indicator used, however, the results cannot be aggregated to compare trends across different sectors if there is inconsistency in the metrics used to quantify use. The indicators could be harmonised if there was open access to the raw data or a unified database, however neither are currently available in Ireland.

To allow for robust comparisons across countries, recommendations for the most suitable metrics to use when quantifying country-level AMU to incorporate usage across all species should be considered. Collineau and colleagues published a review on the selection of appropriate indicators for the quantification of AMU in humans and animals [42]. This review highlights the challenge of making comparisons between populations; the differences in AM products, treatment protocols and the population at risk hamper the comparability of any data collected [42]. Collineau and colleagues recommend the preferred use of dose and course metrics for country-level comparisons, with sector-level mg/PCU as an acceptable alternative. Mills and colleagues suggested that country-specific versions of the daily dose and course metrics would improve representativeness but note that tailoring the metrics may prove impractical in the short-term and the current standardised ESVAC dose and course metrics (DDDvet and DCDvet) are a viable alternative [43]. Currently overall mg/PCU is the metric used by the EMA in their annual reports on veterinary AMU, and it is also used by the European Food Safety Authority (EFSA) and European Centre for Disease Control (ECDC) to monitor AMU and AMR in food-producing animals [44]. However, care should be taken when using mg/PCU for country-level comparisons, as it is heavily influenced by the relative size of different sectors in terms of biomass and use within each sector [45]. Sector-level values of mg/PCU would allow for cross-species comparisons and more accurate comparisons at country-level.

The latest ESVAC report published by the EMA in 2019, describes trends in AM sales for all food-producing species, expressed in mg/PCU, for 25 EU member states from 2011 to 2017 [33]. As mentioned, country-level comparisons using overall mg/PCU should be interpreted with caution, given the size differences of farming sectors in terms of biomass and differences in use within each sector. Ireland has shown comparatively low levels of overall usage, 46.6 mg/PCU, however research in the pig sector has shown AMU levels of 161.9 mg/PCU [35], which is not reflected in the overall mg/PCU as the pig sector accounts for a small percentage of total biomass [46]. Ireland’s predominant livestock sector is cattle, where AMU is typically low, therefore country-level mg/PCU will be favourable. Overall usage has appeared consistent in Ireland during this period (2011–17), however, within-country temporal trends in overall mg/PCU will be influenced by fluctuations in the national herd size. The increase in the size of the cattle herd (in terms of biomass) accompanied by a flatline trend in overall use may actually represent an increase in mg/PCU in the non-cattle sectors. Accepting the issues with mg/PCU as a metric for country-level comparisons, some useful interpretations can be taken from the ESVAC report. The report shows that overall sales of veterinary AM fell by more than 32% between 2011 and 2017, with some of the largest reductions in countries with the highest usage initially [33]. However, usage has appeared consistent in Ireland whilst declining in other countries. For example, the UK, have decreased their AMU, in terms of mg/PCU, by almost 50% in the last 5 years, with reductions in each sector’s mg/PCU [14]. The sales figures suggest Ireland has fallen behind in efforts to reduce global veterinary AMU and progress made in member states highlights the need for change in Ireland. The overall decline in sales has been achieved through the introduction of targeted AMU reduction strategies, including among others, increased monitoring of use, benchmarking, restrictions on use and the introduction of national reduction targets [33]. Ireland’s National Action Plan on AMR (*i*NAP) will be revised in 2020, which provides an opportunity to learn from the success of other EU member states and incorporate improved reduction strategies into the next stages of the plan.

**Fig. 4:**
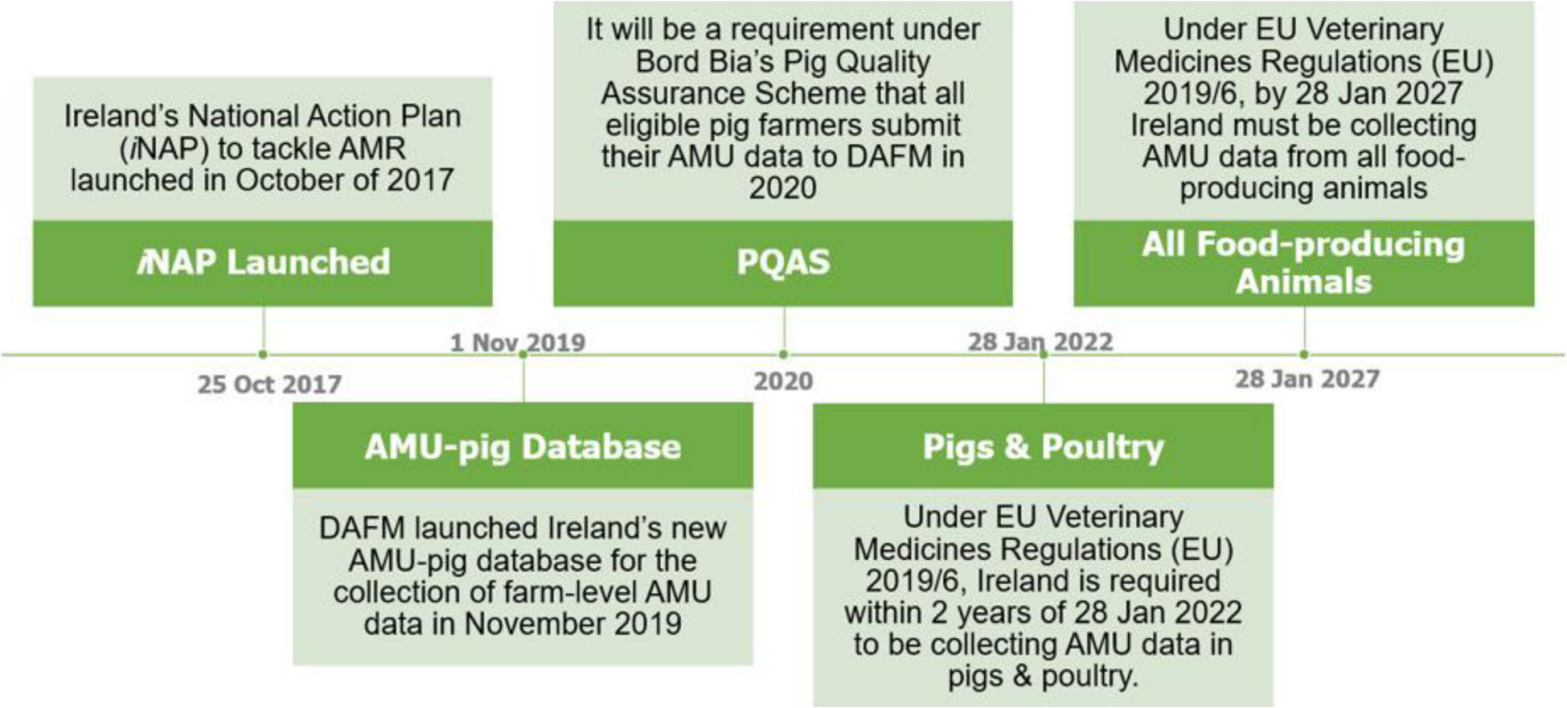
Timeline of events in the collection of AMU data in Ireland

The ability for Ireland to reduce national veterinary AMU will be greatly increased if the monitoring of AMU can be improved. The sector-level data collected in surveillance studies has provided valuable information on AMU in farmed animals albeit on a small scale. To monitor AMU at a larger scale in the pig industry, DAFM have introduced an AM data collection system, ‘AMU Pig’, for commercial pig herds in Ireland, which will report usage in mg/PCU [47]. Approximately 300–400 commercial pig herds (herds slaughtering > 200 pigs per annum) were invited to register for the system, in which farmers will input their AMU quarterly. Currently the system is voluntary, but it will become a requirement for participation in the Bord Bia Pig Quality Assurance Scheme (PQAS) in 2020 and a requirement by EU law in January 2022. Within the poultry industry, DAFM is currently working to produce a national AMU figure for the broiler sector [39]. The layer sector will also need to be considered to provide a comprehensive picture of AMU in the Irish poultry industry. Additionally, the implementation of new EU legislation will result in the collection of AMU data in the less intensive livestock industries, such as dairy, beef and sheep. Figure 4 provides a timeline of events in AMU data collection in the animal health sector in Ireland.

The development of AMU surveillance systems will allow for national use to be monitored and reported. In addition to monitoring trends in AMU at both sector and national level, the availability of usage data will allow Ireland to benchmark farms and veterinarians. Benchmarking has proven to be useful in reducing AMU in other EU member states, such as the Netherlands [13] and Denmark [10]. However, while the collection of AMU data will be valuable for monitoring progress, it comes with its own set of challenges. DAFM anticipate that AMU data from all food-producing animals except pigs will initially be gathered from the veterinarians prescribing the medicines [48]. While prescription data provides a more comprehensive picture of AMU than sales data, there is a possibility of over-reporting as the quantities used by the farmer, the end-user of the product, may be different from that prescribed. The quantification of AMU from prescription data may also be challenging in sectors with traditionally low veterinary involvement, such as sheep. The obtainment of accurate farm-level usage records would be ideal, however the feasibility of obtaining robust and accurate usage records from all farms in Ireland is uncertain. Farmers are required to record usage data. However, maintaining accurate records requires commitment from all those working with livestock, and without any incentives, farmers may lack the motivation to report use resulting in underreporting.

This review provides an insight into current AMU in animal production in the Republic of Ireland and highlights the need for robust AMU data collection across all livestock sectors. The standardisation of data collection methods will allow for comparison within and between species. AMU data collection systems will be developed in the future to ensure Ireland complies with EU regulations however there will be challenges in collecting robust and accurate data. In the short term, smaller surveillance studies would be beneficial to build on knowledge of AMU in food-producing animals, especially in less intensive industries such as beef and sheep, where information is currently lacking.

## Conclusion

The development of AMR is a continuing threat for humans and animals; thus it will be important to monitor AMU and AMR at practice and farm-level across all production sectors. This review has highlighted significant gaps in the knowledge of AMU in food-producing animals in the Republic of Ireland. Farm-level usage data will provide insight into the trends of use and allow for targeted interventions. The efficacy of reduction strategies will be improved if they can be targeted at specific behaviours, patterns of use and specific high usage farms. While progress has been made in the pig and poultry sectors, the less intensive industries such as the beef and sheep sectors, have a long way to go with regards to providing a comprehensive overview of AMU, progress cannot be demonstrated in the absence of data collection.

## Data Availability

Not applicable.

## Abbreviations

AM: Antimicrobial
AMR: Antimicrobial resistance
AMU: Antimicrobial use
AMURAP: Antimicrobial Use and Resistance in Animal Production
AMEG: Antimicrobial Advice Ad Hoc Expert Group
CIA: Critically Important Antibiotics
DAFM: Department of Agriculture, Food & the Marine
DCDvet: Defined Course Doses for animals
DDDvet: Defined Daily Doses for animals
DCT: Dry Cow Therapy; ECDC: European Centre for Disease Control
EFSA: European Food Safety Authority
ESVAC: European Surveillance of Veterinary Antimicrobial Consumption
EMA: European Medicines Agency
EU: European Union
HPRA: Health Products Regulatory Authority
iNAP: Ireland’s National Action Plan on Antimicrobial Resistance
LT: Lactation Therapy
PQAS: Pig Quality Assurance Scheme
PCU: Population correction unit
SDCT: Selective Dry Cow Therapy
TI: Treatment Incidence
UK: United Kingdom
WHO: World Health Organisation
